# A Novel Optimal Configuration form Redundant MEMS Inertial Sensors Based on the Orthogonal Rotation Method

**DOI:** 10.3390/s140813661

**Published:** 2014-07-29

**Authors:** Jianhua Cheng, Jinlu Dong, Rene Jr. Landry, Daidai Chen

**Affiliations:** 1 Marine Navigation Research Institute, College of Automation, Harbin Engineering University, Harbin 150001, China; E-Mails: ins_cheng@163.com (J.C.); ins_dai@163.com (D.C.); 2 LASSENA Laboratory, Ecole de Technologie Superieure, 1100 Notre-Dame Street West, Montreal, QC H3C 1K3, Canada; E-Mail: Rene.Landry@etsmtl.ca

**Keywords:** MEMS, redundancy configuration, reliability, navigation accuracy, FDI

## Abstract

In order to improve the accuracy and reliability of micro-electro mechanical systems (MEMS) navigation systems, an orthogonal rotation method-based nine-gyro redundant MEMS configuration is presented. By analyzing the accuracy and reliability characteristics of an inertial navigation system (INS), criteria for redundant configuration design are introduced. Then the orthogonal rotation configuration is formed through a two-rotation of a set of orthogonal inertial sensors around a space vector. A feasible installation method is given for the real engineering realization of this proposed configuration. The performances of the novel configuration and another six configurations are comprehensively compared and analyzed. Simulation and experimentation are also conducted, and the results show that the orthogonal rotation configuration has the best reliability, accuracy and fault detection and isolation (FDI) performance when the number of gyros is nine.

## 1. Introduction

Inertial measurement units (IMUs) composed by MEMS inertial sensors exhibit a number of advantages such as strong-autonomy, small-volume, light weight, low-cost and good impact resistance. Due to these excellent characteristics, MEMS IMUs have become a hot topic in the inertial navigation field in recent years [1–4]. One of their most innovative applications is to promote the development of space systems and the field of aircraft, such as the application of inertial sensors to spacecraft navigation systems. In order to ensure the precise guidance of spacecraft, higher accuracy and reliability are demanded of IMUs [5]. As for the MEMS inertial sensors, their low-accuracy characteristics have greatly restricted their performance and applications in high-accuracy fields [6,7].

The redundant configuration technique appears to be the most mainstream method for improving the accuracy and reliability of INS, in which the configuration of the system is meticulously designed and the number of sensors is reasonably increased. By assembling a set of inertial sensors with a certain designed configuration, the redundancy of each axis in the navigation framework can be efficiently improved, as well as the accuracy of the whole IMU, because the IMU can make full use of the redundant observation data of these sensors [8,9]. One of the typical applications for that are the redundant systems of the Litton and Honeywell corporations. Litton [10] designed a regular tetrahedron configuration IMU, in which there were one two-axis gyro and two single-axis accelerometers on each surface of the configuration. According to this scheme, we can simplify the measurement equations and improve the interchangeability of sensors. Honeywell [10,11] developed a redundant IMU with six laser gyros and six accelerometers, which could dramatically improve the reliability of the entire system. It has been successfully applied in the Boeing 777 aircraft. Wang *et al.* [12] presented an octadecahedron scheme using nine gyros. The reliability of this scheme is equivalent to six parallel sets of non-redundant IMUs, and its mean time between failures (MTBF) is 1.4 times than that of the regular dodecahedron scheme. However, the sensitive axes of three groups of gyros are coplanar, which restricts the performance of this scheme. It is also too complex for actual installation. Li *et al.* [13] proposed a nine-gyro-four-axis redundant IMU with nine sensors installed along four axes, in which three axes are orthogonal and one axis is skewed. The gyros placed on the skewed axis are used to monitor the data of the orthogonal gyros in real-time. Once a failure in any gyro on the orthogonal axis occurs, the available navigation information can be reckoned by the data of the skewed gyros, so the scheme has a good navigation performance even with a failure condition of the orthogonal gyros. However, compared with other schemes with the same number of sensors, its reliability appears lower as the skewed gyros do not provide measurement data for true navigation calculations.

For the purpose of further enhancing the performance of MEMS IMU, a novel nine-gyro redundant scheme based on orthogonal rotation principle is proposed. This configuration is obtained through a two-rotation from a basic orthogonal configuration. From the navigational viewpoint, we provide the index function of navigation accuracy for redundant configurations. Seven different schemes are investigated, and their reliability indexes are calculated and analyzed. Theoretical analysis and verification based on generalized likelihood test (GLT) method proved the new scheme can greatly improve the accuracy and reliability of MEMS IMU simultaneously. In addition, a T-type structure is designed for simplifying the machining operation process and making this scheme easier to implement for engineering applications [14,15]. Considering the fault detection and isolation (FDI) performance and the reliability, the proposed orthogonal rotation configuration is the optimal scheme when the number of gyros is nine. An experimentation system is established to verify the FDI characteristics for the redundant system under vibration conditions.

The article is organized as follows: Section 2 analyzes the accuracy and reliability of INS. In Section 3, the navigation algorithm of the redundant scheme is discussed first, and then the accuracy and reliability index functions for redundant INS are given. And Section 4 focuses on the optimal redundant nine-gyro configuration based on the orthogonal rotation method. The accuracy and reliability of a total of seven different configurations are compared in Section 5. For the FDI performance, simulation and experimentation of the proposed scheme and the octadecahedron scheme are given in Sections 6 and 7, respectively. Finally, Section 8 gives the conclusions.

## 2. Accuracy and Reliability Analysis of INS

### 2.1. Accuracy Analysis of INS

Among the errors of MEMS inertial sensors, the constant error, scale factor error and installation error can be measured and compensated mostly by calibration tests [16,17]. However, it seems very hard to accurately compensate the random error, because it is too difficult to obtain an accurate error model [18]. As a result, random noise errors are one of the key error sources which dominate navigation accuracy.

By applying random noise to a second order linear oscillator, the effect of random noise on the velocity and position accuracy of INS can be analyzed. At second order linear damped oscillator [19] can be described as:

(1) $$F(s)=\frac{\omega_{0}}{s^{2}+2{\xi \omega }_{0}s+\omega_{0}^{2}}$$

where ξ is the damping coefficient and ω_0_ is the oscillation frequency.

When the INS works under an undamped state, and the input of oscillator is Gaussian white noise, and the root mean square (RMS) of the sensor output signal can be expressed as follows:

(2) $$\begin{array}{cc} \bar{e_{0}^{2}(t)}=\frac{\pi P}{2\omega_{0}}\left(\omega_{0}t-\frac{1}{2}\sin\omega_{0}t\right), & \xi =0 \end{array}$$

where *P* is the average power of the input signal.

**Figure 1.**
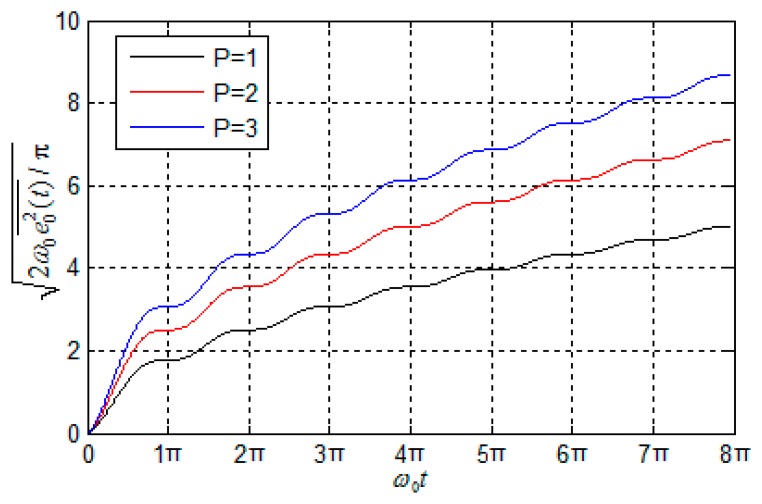
Curves of INS error excited by different random noise.

In order to investigate the influence of the system input on the output, different average powers of input signals are given, and the resulting output curves of $\sqrt{2\omega_{0}\bar{e_{0}^{2}(t)}/\pi }$ are shown in Figure 1. From this figure, the RMS of INS errors excited by random noise accumulates over time. What is more, the greater the random noise power value is, the faster the navigation errors accumulate. Therefore, inhibiting the random noise is quite an effective technique for improving the accuracy of a navigation system.

### 2.2. Reliability Analysis of INS

High-reliability inertial sensors are the basic foundation for IMUs to complete guidance and navigation tasks successfully. Generally, the reliability expression of a single inertial sensor (*i.e.*, single-axis gyro) can be described as [20,21]:

(3) $$R(t)=e^{-\lambda t}$$

where λ is the failure rate.

Consider the basic IMU situation where a sensor failure occurs equally and independently, the reliability expression of non-redundant IMU can be written as:

(4) $$R_{\text{IMU}}={\left(R(t)\right)}^{3}=e^{-3\lambda t}$$

From Equations (3) and (4), the reliability of the IMU is directly related to the reliability of a single inertial sensor. That means that only if the single inertial sensor has a high reliability a non-redundant system will exhibit a high reliability.

## 3. Criteria for Redundant Scheme Design

### 3.1. Navigation Algorithm of Redundant Scheme

In conventional schemes, the IMU is rigidly mounted along with the body coordinate system (*ox_b_y_b_z_b_*). This guarantees that the inertial sensor can efficiently measure the linear and angular motion information of the body coordinate system with respect to the inertial space. However, for the redundant scheme, the inertial sensors may be non-orthogonal and cannot ensure all gyro-sensitive axes coincide with the body coordinate system. In order to facilitate the navigation calculation, it is necessary to resolve the measurements of gyros in the body coordinate system.

**Figure 2.**
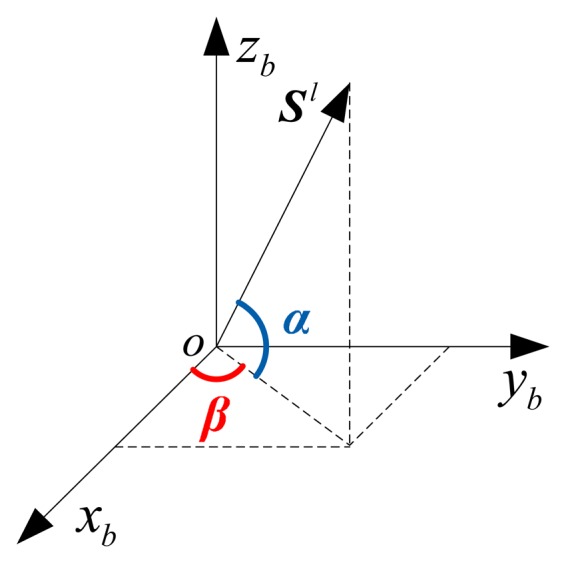
Projection of gyro sensitive axis in the body coordinate system.

The body coordinate system *ox_b_y_b_z_b_* is shown in Figure 2, where *ox_b_* and *oy_b_* are the lateral and longitudinal axes along the direction of carrier, respectively. *ox_b_*, *oy_b_* and *oz_b_* constitute a right-handed Cartesian coordinate system.

Assume that the output of *l*th gyro in a redundant IMU is ***S****^l^*, then the angle between the sensitive axis of gyro and the plane *ox_b_y_b_* is α, and the angle between the projection of ***S****^l^* on the plane *ox_b_y_b_* and the axis *ox_b_* is β. According to Figure 2, the output of gyro can be derived and expressed in the vector form [22]:

(5) $${\text{S}}^{l}=\cos\alpha \cos\beta \text{i}+\cos\alpha \sin\beta \text{j}+\sin\alpha \text{k}={\text{S}}_{1}^{l}\text{i}+{\text{S}}_{2}^{l}\text{j}+{\text{S}}_{3}^{l}\text{k}$$

where, ***i***, ***j*** and ***k*** are three unit vectors along *ox_b_*, *oy_b_* and *oz_b_* of the body coordinate system, respectively.

For the redundant scheme with the total number of gyros is *n* (*n* ≥ *l*), the measurement equation of *n* gyros can be described as follows:

(6) $$\left[\begin{array}{c} m_{1}\\ m_{2}\\ \vdots \\ m_{n} \end{array}\right]=\left[\begin{array}{ccc} S_{1}^{1} & S_{2}^{1} & S_{3}^{1}\\ S_{1}^{2} & S_{2}^{2} & S_{3}^{2}\\ \vdots & \vdots & \vdots \\ S_{1}^{n} & S_{2}^{n} & S_{3}^{n} \end{array}\right]\cdot \left[\begin{array}{c} \omega_{x}\\ \omega_{y}\\ \omega_{z} \end{array}\right]+\left[\begin{array}{c} \eta_{1}\\ \eta_{2}\\ \vdots \\ \eta_{n} \end{array}\right]$$

where, *m_i_* is the output of the *i*th gyro (*i* = 1,2,…,*n*); ω*_x_*, ω*_y_* and ω*_z_* are three angular rates along *ox_b_*, *oy_b_* and *oz_b_* of the body coordinate system, respectively; η*_i_* is the measurement error.

Equation (6) can also be represented as the following vector form:

(7) m=Hω+η

where, ***m*** is the measurement vector; ***H*** is the measurement matrix; ω is the angular rate vector; η is the measurement noise vector.

Assume the measurement noise is a Gaussian white noise with a zero-mean value and standard deviation $\sigma_{\eta }^{2}$, we have:

(8) $$\begin{array}{cc} E(\text{η})=0; & E(\text{η}{\text{η}}^{T})=\sigma_{\eta }^{2}{\text{I}}_{n} \end{array}$$

where, ***I****_n_* is a *n*-dimension identity matrix.

Based on the theory of linear weighted minimum variance, the estimated value of the navigation input ω can be expressed as:

(9) $$\hat{\omega }={\left({\text{H}}^{T}\text{WH}\right)}^{-1}\text{HWm}$$

where ***W*** is the weighted matrix; ω̂ is the estimated navigation input of the INS, which can be directly used to calculate transform matrix of INS, then the velocity and position can be obtained with the measurement from the accelerometer [10].

### 3.2. Accuracy Index Function of the Redundant Scheme

Equation (9) shows that when the random noise of gyro is confirmed the matrix ***H*** will directly affect the characteristics of ω̂. Therefore, it will be useful to improve the characteristic of ω̂ if the matrix ***H*** is configured reasonably, and consequently the navigation accuracy is improved.

Defining the estimate error of angular rate ω̂ = ω − ω̂ as follows:

(10) $$\tilde{\omega }=\omega -{\left({\text{H}}^{T}\text{WH}\right)}^{-1}\text{HWm}=-{\left({\text{H}}^{T}\text{WH}\right)}^{-1}\text{HW}\eta$$

From Equation (10), the estimate error ω̂ follows normal distribution with zero-mean value, and its variance is:

(11) $$\text{Var}(\tilde{\omega })=E[{\tilde{\omega }\tilde{\omega }}^{T}]={\left({\text{H}}^{T}\text{WH}\right)}^{-1}{\text{H}}^{T}\text{WRWH}{\left({\text{H}}^{T}\text{WH}\right)}^{-1}$$

where, $\text{R}=\text{Var}(\text{η}{\text{η}}^{T}){=\sigma }_{\eta }^{2}{\text{I}}_{n}$.

Equation (11) can be simplified as follows:

(12) $$\text{Var}(\tilde{\omega })={\left({\text{H}}^{T}{\text{R}}^{-1}\text{H}\right)}^{-1}=\sigma_{\eta }^{2}{\left({\text{H}}^{T}\text{H}\right)}^{-1}$$

The mean square error of estimate error can be represented by the following normalized form:

(13) $$\sigma_{\omega }^{2}{\text{I}}_{3}=\frac{\text{Var}(\tilde{\omega })}{\sigma_{\eta }^{2}}={\left({\text{H}}^{T}\text{H}\right)}^{-1}$$

Then, we could obtain the probability density function of the estimate error as follows [23]:

(14) $$f(\eta )=\frac{1}{{\left(2\pi \right)}^{n/2}{\left|\text{C}\right|}^{1/2}}\exp\{-\eta^{T}\eta /\left(2\text{C}\right)\}$$

where, ***C*** = (***H***^*T*^ ***H***)^−1^. The trajectory of η is determined by amplification factor *K* = η*^T^*η/***C***.

Equation (14) actually represents a family of ellipsoids. For each value of *K*, there will be a corresponding ellipsoid. The bulk of this ellipsoid is:

(15) $$V_{\text{bulk}}=\frac{4}{3}K^{3/2}\pi \sqrt{\left|\text{C}\right|}$$

From Equation (15), we could know that if *K* keeps a constant value the bulk of the ellipsoid depends on the determinant value of matrix ***C***. A smaller bulk of the ellipsoid means a smaller estimate error of the system as well as a better navigational accuracy of the IMU.

According to Equations (13) and (15), the index function of navigation accuracy is obtained as follows:

(16) $$\Phi =\sqrt{\left|\text{C}\right|}={\left\{\det({\text{H}}^{T}\text{H})\right\}}^{-1/2}$$

### 3.3. Reliability Index Function of Redundant Configuration

MTBF is the average operation time between inherent failures of a single sensor or a system, which can be calculated as the arithmetic mean time between failures of a system. It is commonly selected as a reliability index of a sensor or product, which could effectively show the quality of the product with respect to the operation time. According to Equation (3), the MTBF of single gyro can be expressed as:

(17) $${\text{MTBF}}_{\text{gyro}}=\int_{0}^{\infty }R(t)\text{dt}=\frac{1}{\lambda }$$

The corresponding MTBF of non-redundant IMU is:

(18) $${\text{MTBF}}_{\text{IMU}}=\int_{0}^{\infty }e^{-3\lambda t}\text{dt}=\frac{1}{3\lambda }$$

From Equations (17) and (18), the reliability of a non-redundant IMU is very low, only 1/3 of the reliability of a single gyro.

## 4. Nine-Gyro Redundant Configuration Based on the Orthogonal Rotation Method

The nine gyros of the proposed redundant IMU scheme can be described as *m*_1_, *m*_2_, …, *m*_9_ sequentially. For the convenience of the scheme design, all gyros are divided into three groups (*m*_1_, *m*_4_, *m*_7_), (*m*_2_, *m*_5_, *m*_8_) and (*m*_3_, *m*_6_, *m*_9_). The sensitive axes of gyro in each group are orthogonal with each other.

The directions of sensitive axes for all gyros in the new redundant configuration based on orthogonal rotation method are formed as follows:

(1) The sensitive axes of *m*_1_, *m*_4_, *m*_7_ coincide with *ox_b_*, *oy_b_* and *oz_b_* of the body coordinate system;
(2) Define a new vector ***S*** in the body coordinate system, and the angles between the vector ***S*** and each axis of the body coordinate system are equally (the angle is 54.736°). Assume that *m*_2_, *m*_5_, *m*_8_ are located on the three axes of the body coordinate system at the initial time, and we rotate them counterclockwise around ***S*** by 40°, then we obtain a frame and the sensitive axes of *m*_2_, *m*_5_, *m*_8_ coincide with this frame axis directions, respectively;
(3) Similarly, we continue to rotate them around ***S*** with 40° counterclockwise, and we could obtain the frame, and the sensitive axes of *m*_3_, *m*_6_, *m*_9_ coincide with the directions of this frame axes, respectively.

**Figure 3.**
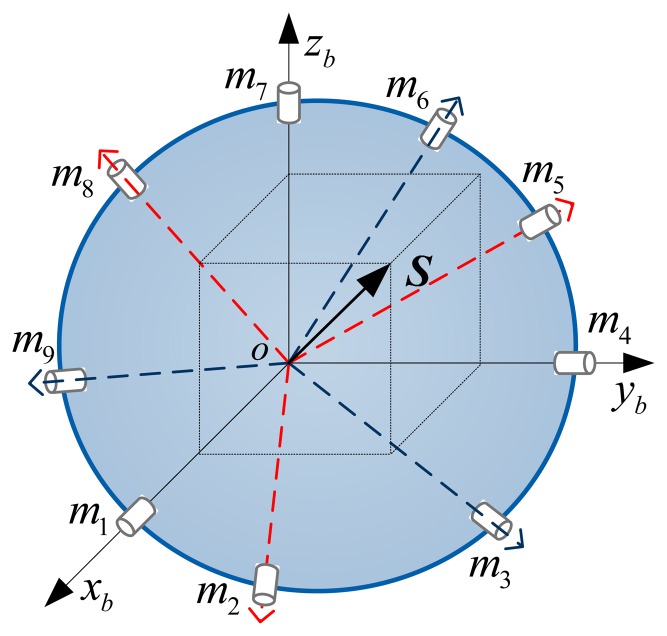
The nine-gyro redundant configuration based on orthogonal rotation method.

According to the rotation strategy above, the sensitive axes gyros in the proposed scheme are shown in Figure 3.

By calculating (see Appendix A), the angles among sensitive axes of gyros have the following regularity:

(1) The angle between any two adjacent sensitive axes of gyros is α = 32.43°, such as *m*_1_ and *m*_2_, *m*_1_ and *m*_9_;
(2) The angle between any two interval sensitive axes of gyros is β = 63.32°, such as *m*_4_ and *m*_6_, *m*_7_ and *m*_9_;
(3) The angle between any two opposite sensitive axes of gyros is θ = 107.05°, such as *m*_1_ and *m*_5_, *m*_4_ and *m*_8_, *m*_7_ and *m*_2_.

According to Figure 3 and the analysis of angle regularity, the measurement matrix ***H*** can be expressed as:

$$\text{H}={\left[\begin{array}{lllllllll} 1 & \cos\alpha & \cos\beta & 0 & \cos\theta & \cos\theta & 0 & \cos\beta & \cos\alpha \\ 0 & \cos\beta & \cos\alpha & 1 & \cos\alpha & \cos\beta & 0 & \cos\theta & \cos\theta \\ 0 & \cos\theta & \cos\theta & 0 & \cos\beta & \cos\alpha & 1 & \cos\alpha & \cos\beta \end{array}\right]}^{T}$$

As shown in the measurement matrix, the angular rate along each axis of this redundant configuration can be measured by seven gyros. Therefore, its reliability is equivalent to seven parallel sets of non-redundant IMUs.

Besides the theoretical design, structural design and installation may be another crucial problem for its real application. There are quite a number of gyros that need to be mounted on an irregular or regular frame. To realize the configuration shown in Figure 3, all gyros should be re-arranged to three new groups (*m*_1_, *m*_2_, *m*_3_), (*m*_4_, *m*_5_, *m*_6_) and (*m*_7_, *m*_8_, *m*_9_). The angles of the gyros in each new group have a new regularity:

(1) The angles between any adjacent sensitive axes of gyros are α = 32.43°, such as *m*_1_ and *m*_2_, *m*_2_ and *m*_3_, *m*_4_ and *m*_5_, *m*_5_ and *m*_6_, *m*_7_ and *m*_8_, *m*_8_ and *m*_9_;
(2) The angles between any two interval sensitive axes of gyros are β = 63.32°, such as *m*_1_ and *m*_3_, *m*_4_ and *m*_6_, *m*_7_ and *m*_9_.

The new three groups can be orthogonally installed more conveniently. Such a method can meet the requirements of (*m*_1_, *m*_4_, *m*_7_), (*m*_2_, *m*_5_, *m*_8_) and (*m*_3_, *m*_6_, *m*_9_) in Figure 3, which could simplify the construction process and make it easier for engineering realization.

## 5. Comparison of Schemes on Accuracy and Reliability

**Figure 4.**
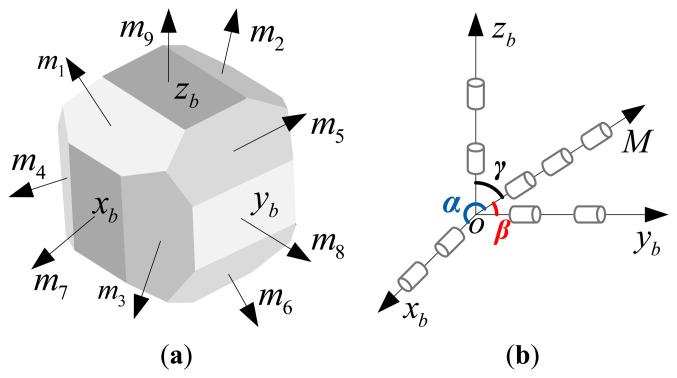
(**a**) Octadecahedron configuration; (**b**) Nine-gyroscope-four-axis configuration.

To verify the navigation accuracy and reliability of the proposed scheme, a comparative analysis is conducted with other excellent schemes. [10,12,13] present four schemes: tetrahedron, dodecahedron, octadecahedron and nine-gyro-four-axis configurations. The configurations of the octadecahedron and nine-gyro-four-axis schemes in [12,13] are shown in Figure 4a,b, respectively.

The measurement matrix of the octadecahedron configuration is:

$${\text{H}}_{1}={\left[\begin{array}{ccccccccc} S & -S & C & C & 0 & 0 & 1 & 0 & 0\\ 0 & 0 & S & -S & C & C & 0 & 1 & 0\\ C & C & 0 & 0 & S & -S & 0 & 0 & 1 \end{array}\right]}^{T}$$

where, *S* = sin α, *C* = cos α, α = 31.70°, and the measurement matrix of the nine-gyro-four-axis configuration is:

$${\text{H}}_{2}={\left[\begin{array}{ccccccccc} 1 & 1 & 0 & 0 & 0 & 0 & \cos\alpha & \cos\alpha & \cos\alpha \\ 0 & 0 & 1 & 1 & 0 & 0 & \cos\beta & \cos\beta & \cos\beta \\ 0 & 0 & 0 & 0 & 1 & 1 & \cos\gamma & \cos\gamma & \cos\gamma \end{array}\right]}^{T}$$

where, α = β = γ = 54.74°.

### 5.1. Comparison of Scheme Accuracy

According to Equation (16), the navigation accuracy index of the new redundant scheme can be calculated as:

$$\Phi =\sqrt{\left|\text{C}\right|}={\left\{\det({\text{H}}^{T}\text{H})\right\}}^{-1/2}=0.1925$$

The navigation accuracy index of the other redundant schemes can be calculated using the same equation, giving the results shown in Table 1.

As shown in Table 1, the accuracy index values become successively smaller from top to bottom. Therefore, their corresponding navigation accuracies of these schemes grow higher from top to bottom. In the case of nine gyros, of the three schemes on bottom, the navigation accuracy of the nine-gyro-four-axis scheme is the worst, and the navigation index values of the orthogonal rotation and octadecahedron schemes are equivalent, whereby both can achieve good performance.

### 5.2. Comparison of Scheme Reliability

For the redundant scheme shown in Figure 3, any three gyros of the IMU are not coplanar. The reliability of a redundant system can be calculated as follows:

(19) $$R(9)=\sum_{n=3}^{9}C_{9}^{n}R^{n}{\left(1-R\right)}^{m-n}$$

where, $C_{9}^{n}=\frac{9!}{n!(9-n)!}$.

The corresponding MTBF is:

(20) $${\text{MTBF}}_{\text{Orthogonal}-\text{rotation}}=\int_{0}^{\infty }R(9)\text{dt}=\frac{3349}{2520\lambda }$$

For the redundant octadecahedron configuration, *m*_1_, *m*_2_ and *m*_9_ are coplanar, as well as *m*_3_, *m*_4_, *m*_7_ and *m*_5_, *m*_6_, *m*_8_, and the normals of these three planes are perpendicular with each other, so the corresponding MTBF is given by:

$${\text{MTBF}}_{\;\text{Octadecahedron}}=\frac{6377}{5000\lambda }$$

For the redundant nine-gyro-four-axis configuration, three gyros on the skewed axis only play a monitoring role. The corresponding MTBF is given by:

$${\text{MTBF}}_{\text{Nine}-\text{gyro}-\text{four}-\text{axis}}=\frac{261}{500\lambda }$$

Assuming that the total work time of a system is one year, and the MTBF of a single-axis gyro is 20,000 h, the reliabilities of different redundant configurations are shown in Table 2.

**Figure 5.**
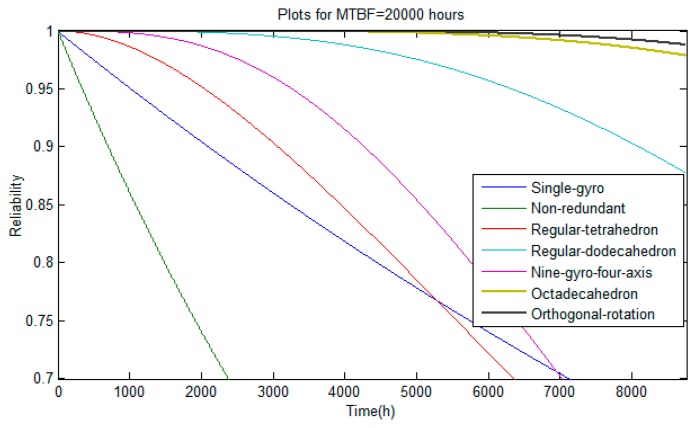
Reliability curves of seven different schemes.

As for the reliability of schemes, the greater the reliability index value is, the better reliability performance it shows, which is different from the accuracy index. As shown in Table 2, the reliability index value of the non-redundant orthogonal configuration is very small. By contrast, the redundant schemes can efficiently improve the reliability of schemes, especially the last two ones. The proposed scheme in this paper exhibits the best reliability performance among these seven schemes. The reliability curves are shown in Figure 5. As can be seen from Figure 5:

(1) The reliability of a system can be improved by increasing the number of gyros, however, the effect will become less obvious as the number of gyros increases;
(2) For the octadecahedron and orthogonal rotation configurations, their reliabilities vary very little with time, and always remain higher than 0.95;
(3) Over the entire time interval, the reliability of orthogonal rotation configuration is always the best.

## 6. Analysis of Fault-Detection and Isolation

### 6.1. Fault-Detection and Isolation Equations

GLT is an effective FDI method based on parity space theory. By structuring a parity matrix, GLT can check the singularity of system mathematical models to achieve the FDI. To make sure the redundant scheme an excellent performance, it should have a high fault isolation rate together with a low false alarm rate.

For the measurement equation shown in Equation (7), the parity equations of non-fault and fault are given as follows, respectively:

(21) $$\{\begin{array}{l} {\text{P}}_{\text{non}-\text{fault}}=\text{Vm}=\text{V}\eta \\ {\text{P}}_{\text{fault}}=\text{Vm}={\text{Vb}}_{f}+\text{V}\eta \end{array}$$

where, ***V*** is (*n* − 3) × *n* dimensions matrix, which satisfies ***VH*** = 0, ***VV***^*T*^ = ***I***_(_*_n_*_−3)×(_*_n_*_−3)_, and can be acquired by the Potter algorithm [24].

Equation (22) can be used to calculate fault detection function of system, which can judge whether the fault has occurred [25]:

(22) $$FD_{\text{GLT}}={\text{P}}^{T}\text{P}/\sigma_{\eta }^{2}$$

Equation (23) can be used to calculate fault isolation function of system, which can judge which gyro is faulty [25]:

(23) $$FI_{\text{GLT}}(i)={\left({\text{P}}^{T}{\text{v}}_{i}\right)}^{2}/\left(\sigma_{\eta }^{2}{\text{v}}_{i}^{T}{\text{v}}_{i}\right)$$

where, ***v****_i_* is the *i* th column of ***V***.

If Equation (24) is satisfied, it means a failure occurs on the *k* th gyro.

(24) $${\text{FI}}_{\text{GLT}}(k)=\underset{1\leq i\leq n}{\max}\{{\text{FI}}_{\text{GLT}}(i)\}$$

### 6.2. Case of a Single-Gyro Failure

**Figure 6.**
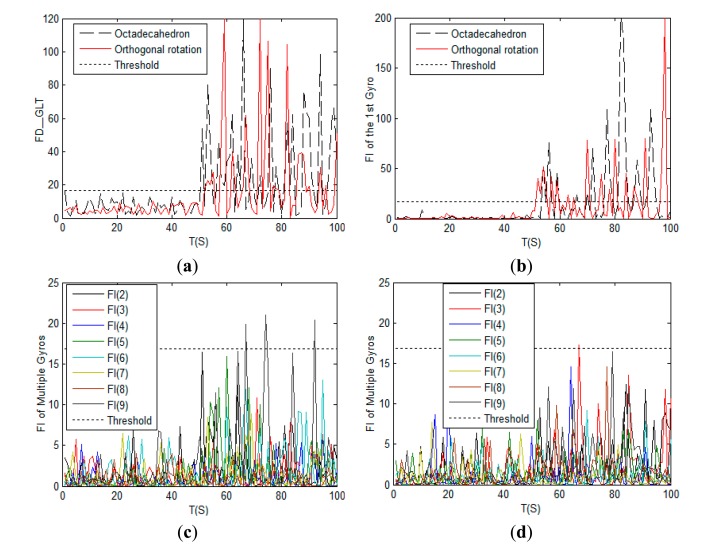
Comparison of single-fault detection and isolation of the octadecahedron and the orthogonal rotation configurations: (**a**) Fault judgment curves; (**b**) Fault detection curves of the 1st gyro; (**c**) Fault detection curves from the 2nd to the 9th gyro of the octadecahedron scheme; (**d**) Fault detection curves from the 2nd to the 9th gyro of the orthogonal rotation scheme.

Simulation parameters are set as: (1) standard deviation of gyro is σ_η_ = 0.5°/h; (2) false alarm rate is α = 0.01, the threshold value *T_D_* is calculated according to α: $T_{D}=\chi_{0.99}^{2}(9-3)=16.8119$ (3) total simulation time is 100 s; (4) at the 51th second a step signal is added on the 1st gyro to simulate a failure, and the signal to noise ratio (SNR) of the faulty signal is 5. The simulation results are shown in Figure 6:

As shown in Figure 6a,b, the GLT method has a nice FDI capacity for both the octadecahedron and the orthogonal rotation configurations. However, for the octadecahedron scheme shown in Figure 6c, its false alarm rate of the 9th gyro is slightly higher than that of the proposed scheme which is shown in Figure 6d.

### 6.3. Case of Double-Gyro Failures

**Figure 7.**
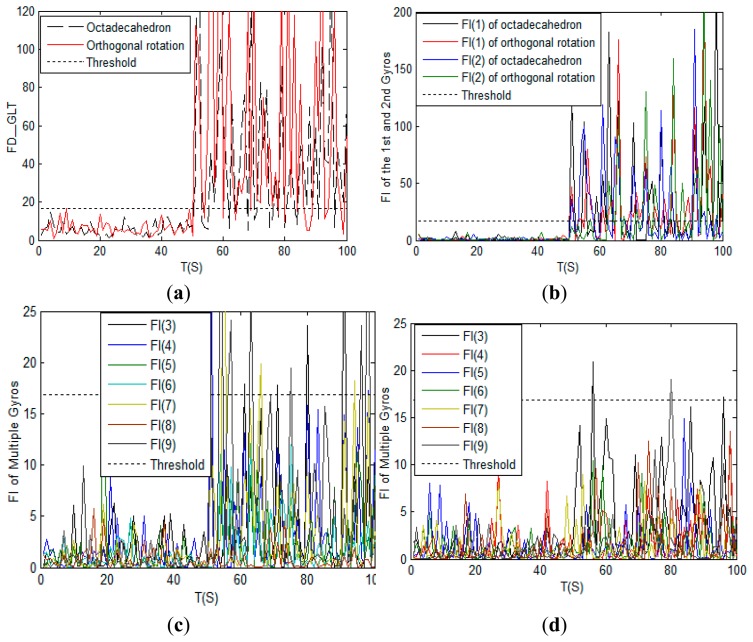
Comparison of double-fault detection and isolation of the octadecahedron and the orthogonal rotation configurations: (**a**) Fault judgment curves; (**b**) Fault detection curves of the 1st and the 2nd gyro; (**c**) Fault detection curves from the 3rd to the 9th of the octadecahedron scheme; (**d**) Fault detection curves from the 3rd to the 9th of the orthogonal rotation scheme.

We add the step fault on the 1st and the 2nd gyro at the 51st s, and the other simulation conditions remain the same. The simulation results are shown in Figure 7.

As shown in Figure 7a, the fault detection function curves are more apparent than those in Figure 6a, and GLT method can still efficiently detect the failures of gyros. From Figure 7d, the false alarm rate of the proposed orthogonal rotation scheme is slightly over the threshold value, and much better than the rate of the octadecahedron scheme.

## 7. Experimentation

**Figure 8.**
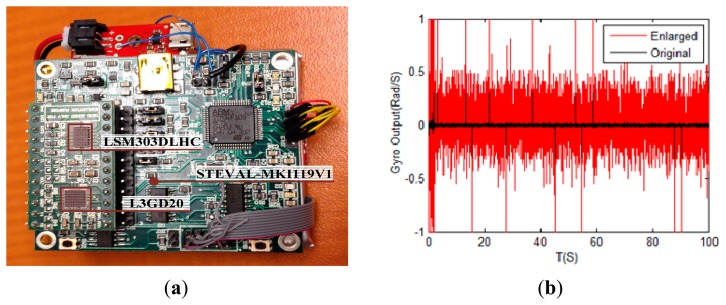
Photograph of the experimentation system and the recorded gyro data. (**a**) The experimentation system; (**b**) The original and enlarged gyro signal.

An experimentation system is constructed to verify the FDI performance for the redundant system, as shown in Figure 8a. Three main components of the system are: (1) L3GD20 (STMicroelectronics, Ottawa, Canada): the MEMS motion sensor includes a three-axis digital output gyroscope [26]; (2) LSM303DLHC (STMicroelectronics, Ottawa, Canada): the ultra-compact high-performance eCompass module includes a 3D accelerometer and 3D magnetometer [27]; (3) STEVAL-MKI119V1 (STMicroelectronics, Ottawa, Canada): eMotion Win8 includes STEVAL-MKI109V2 and STEVAL-MKI108V2 demonstration boards [28], which could be seen as the platform for combining the L3GD20 and LSM303DLHC.

### 7.1. Case of Single-Gyro Failure

Experimentation conditions are setting as: (1) The output of the inertial sensor is firstly processed by subtracting the statistical mean value of MEMS drift, and then the signal is enlarged 30 times for the FDI. The comparison for the original and enlarged output of the Gyro-x is shown in Figure 8b; (2) Total sampling time is 100 s, and add the step fault signal on the 1st gyro at the 51st s, and the SNR of the fault signal is 5.

**Figure 9.**
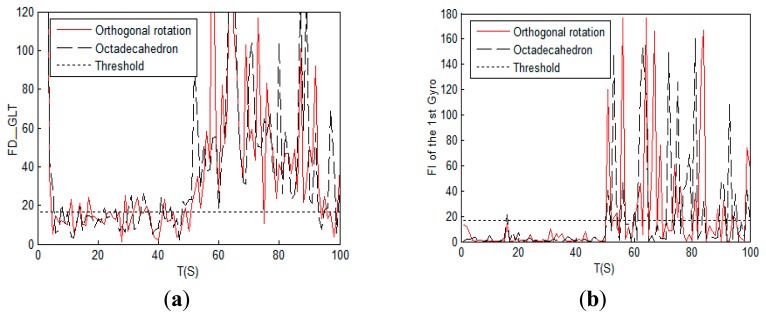
Single-fault detection and isolation of the octadecahedron and the orthogonal rotation configurations: (**a**) Fault judgment curves; (**b**) Fault detection curves of the 1st gyro.

The comparison of fault detection functions and fault isolation performances for the octadecahedron and the orthogonal rotation configurations are shown in Figure 9. As seen in this figure, if one of the gyros in the configurations fails, the GLT method could efficiently detect and locate the fault in both the octadecahedron and orthogonal rotation configurations. Although the fault detection value is greater than the threshold value sometimes, there is an obvious distinction between the no failure and the single-gyro failure situations. As a result, in the case of single gyro fault, the experimentation gives a similar result as the simulation curves in Section 6.1.

### 7.2. Case of Double-Gyro Failure

**Figure 10.**
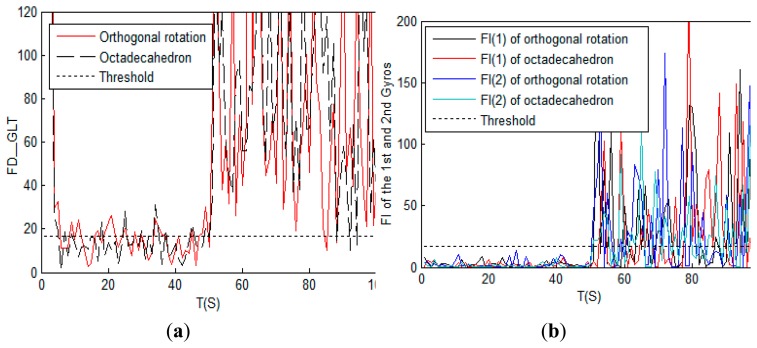
Double-fault detection and isolation of the octadecahedron and the orthogonal rotation configurations: (**a**) Fault judgment curves; (**b**) Fault detection curves of the 1st and the 2nd gyro.

Experimentation conditions are set the same as those in Section 7.2, except there are two gyros undergoing failures under this condition. The comparison of fault detection functions and fault isolation performances for the octadecahedron and the orthogonal rotation configurations is shown in Figure 10.

As shown in Figure 10, under double gyro failure conditions, the octadecahedron and orthogonal rotation configurations are both sensitive to the sensor faults, and the fault could be efficiently detected and accurately positioning by the GLT method. This experimental result shows that the proposed scheme still has excellent performance in a real vibration environment.

## 8. Conclusions

Accuracy and reliability are the most important indexes for a MEMS IMU. This paper puts forward an orthogonal rotation-based nine-gyro redundant MEMS scheme to improve the reliability and accuracy of systems, and a reasonable installation idea is also proposed for the convenience of construction. Simulation and experimentation are conducted and the results verify the effectiveness and FDI performance of the new scheme. The GLT method is also introduced to compare its performance with the octadecahedron scheme in the case of single and double gyros faults. The results show that the new scheme has a good fault-tolerant capability, a low false alarm rate and an excellent performance, even under vibration conditions. The orthogonal rotation configuration has the best comprehensive performance when the number of gyros is nine.

## Figures and Tables

**Table 1.** The value Φ of different redundant schemes.

Configuration	Φ
Regular-tetrahedron	0.6495
Regular-dodecahedron	0.3535
Nine-gyro-four-axis	0.2236
Orthogonal-rotation	0.1925
Octadecahedron	0.1925

**Table 2.** Reliability of different schemes.

Configurations and Number of Gyros	Reliability
Single-gyro	0.6453
Non-redundant	0.2687
Regular-tetrahedron	0.5547
Regular-dodecahedron	0.8774
Nine-gyro-four-axis	0.5585
Octadecahedron	0.9785
Orthogonal-rotation	0.9879

**Figure A1.**
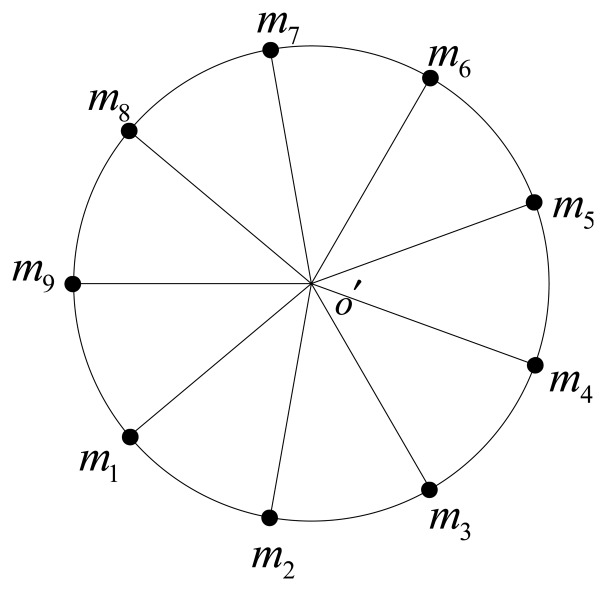
The top view from point *o* to the direction of the vector ***S***.

## Appendix A

Assume that the distances between the points *o*, *o′* and gyros are *a* and *b*, respectively.

So ‖*om*_1_‖ = ‖*om*_2_‖ = ⋯ =‖*om*_9_‖ = *a*; ‖*o′**m*_1_‖ = ‖*o′**m*_2_‖ = ⋯ =‖*o′**m*_9_‖ = *b*.

From Figure 3, the directions of *om*_1_ and *om*_4_ are mutually orthogonal.

Then $\left\|m_{1}m_{4}\right\|=\sqrt{2}a$.

From Figure A1, ∠*m*_1_*o′**m*_4_ = 120°. According to the cosine theorem, we have $b=\frac{\sqrt{6}}{3}a$.

According to ∠*m*_1_*o′**m*_2_ = 40° and the cosine theorem, ‖*m*_1_*m*_2_‖ = 0.5585*a*.

By calculation, cos α = 0.844. The angle between any two adjacent sensing axes of gyros is α = 32.43°.

The values of β and θ can be calculated in the same way, and the angles between any two interval and opposite sensing axes of gyros are β = 63.32° and θ = 107.05°, respectively.
